# Supplementary material for: Alternatively Spliced Landscape of PPARγ mRNA in Podocytes Is Distinct from Adipose Tissue
Source: Cells. 2022 Nov 1;11(21):3455. doi: 10.3390/cells11213455 (PMC9653906; doi:10.3390/cells11213455)
Supplement: Supplementary file 1 [file cells-11-03455-s001.zip › Supplementary Figures.pdf]

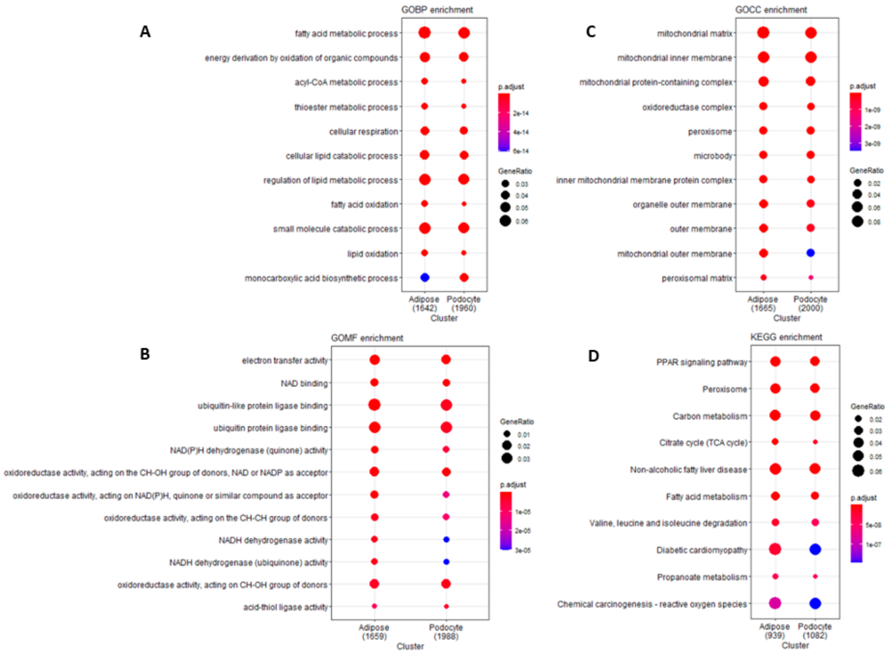

Supplementary Figure S1. ClusterProfiles generated functional enrichment of all PPRE containing genes detectable in adipocytes (1776) or podocytes (2122) and plotted as 10 terms per cell type for (A) biological processes, BP, (B) cellular components, CC, (C) molecular functions, MF, and (D) kyoto encycopedia of genes and genomes, KEGG. The color of the dot indicates the intensity of adj p value (smaller adj p value is more red) and size of the dot indicates the proportion of genes from the term that are present in the cell-specific PPRE containing genes.

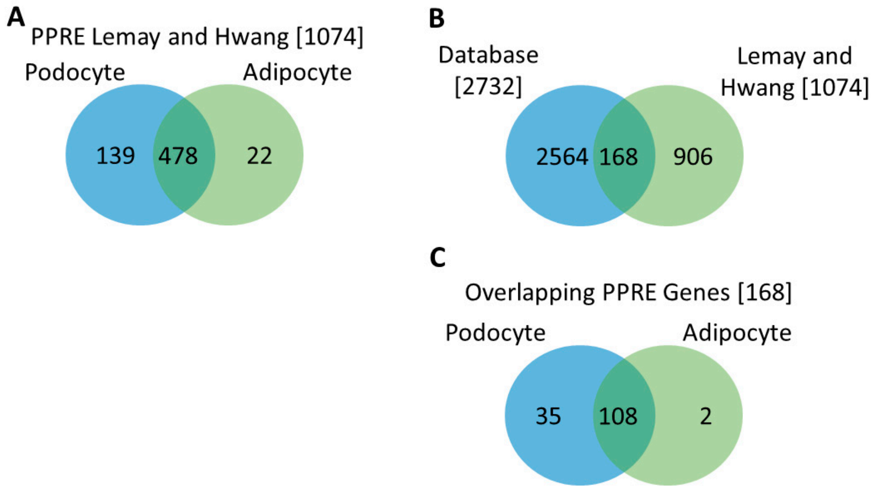

Supplementary Figure S2. PPARγ-Response Element (PPRE) Containing Genes (A) in Podocytes vs Adipocytes. Normalized average log counts of untreated control human differentiated podocyte and adipocyte GEO datasets (GSE124622 and GSE129153, respectively) were cross-referenced against the human PPRE predicted genes described in Lemay and Hwang manuscript. 478 PPRE-containing genes were detectable in both podocyte and adipocyte datasets, 139 were unique to podocytes and 22 unique to adipocytes. (B) PPRE-containing genes from the PPARgene database and Lemay and Hwang. (C) Majority of the overlapping PPRE genes (168) between the PPARgene dataset and Lemay and Hwang manuscript were detected in podocytes and/or adipocytes.
